# A cluster of intracellular parasitic infections among patients on biological DMARDs – the tip of the iceberg?

**DOI:** 10.1093/rap/rky048

**Published:** 2018-11-30

**Authors:** Helena Hammarström, Leif Dotevall, Ann-Marie Calander

**Affiliations:** 1 Department of Infectious Diseases, Sahlgrenska Academy at University of Gothenburg; 2 Department of Infectious Diseases, Sahlgrenska University Hospital, Gothenburg, Sweden; 3 Department of Communicable Disease Control, Region Vastra Gotaland, Sweden; 4 Department of Rheumatology and Inflammation Research, Sahlgrenska Academy at University of Gothenburg, Gothenburg, Sweden

**Key message**

- Increased risk of leishmaniasis in patients on biological DMARDs.

Sir, Patients with rheumatic diseases on immunomodulatory treatment are at increased risk of infections and reactivation of latent infections; thus, screening programmes for tuberculosis and viral hepatitis are executed in most countries before starting treatment with biological drugs. However, the risk for other serious infections in this patient cohort may be greater than recognized so far. A review by Guedes-Barbosa *et al.* [1] suggests an association between an increased risk for leishmaniasis and treatment with TNF-α inhibitors, something we now want to reinforce with our observations. We additionally show that there may be a need to include other biological DMARDs on the awareness list, and we pinpoint some of the pitfalls linked to this neglected infection. Written informed consent was obtained from all patients in this report.

Leishmaniasis is listed by the World Health Organization as a neglected tropical disease with an estimated global prevalence of 12 million cases and an incidence rate of ∼1.3 million cases per year. This vector-borne infection is caused by a protozoan parasite of the genus *Leishmania* and transmitted to humans by phlebotomine sandflies. Leishmaniasis is endemic in South America, Africa, the Middle East, Central Asia and the Mediterranean basin in Europe. The clinical spectrum of leishmaniasis ranges from subclinical infection to a potentially fatal visceral disease characterized by chronic fever, weight loss, hepatosplenomegaly and pancytopenia [2]. In the immunocompetent population it is estimated that only 1 out of every 5–15 infected persons develop symptomatic leishmaniasis [3], and the risk for active and severe disease is much greater among patients with an impaired cell-mediated immune response [4]. The issue of increased risk for leishmaniasis in the era of TNF-α antagonist therapy was recently acknowledged by the European Centre for Disease Prevention and Control [5] and, considering the growing population receiving such treatment, the question undoubtedly deserves further elucidation.

Here, we describe a cluster of seven cases of leishmaniasis among patients from Sweden with rheumatic disease who had all visited the same Leishmania endemic region in Spain. Three Swedish patients treated with TNF-α inhibitors, one with RA and two with PsA, were diagnosed with cutaneous leishmaniasis caused by *Leishmania infantum*. All of them had spent 1 month at a rehabilitation unit in the province of Alicante, Spain before onset of disease. Leishmaniasis is endemic in Spain, where dogs constitute the main animal reservoir for *L. infantum* [6]. The touristic province of Alicante has previously been reported as a high-endemic spot for leishmaniasis, with an estimation of >50% prevalence of subclinical leishmaniasis in adults residing in the province [7]. Being a non-endemic country with <0.4 cases of imported leishmaniasis per 100 000 persons/year [8], leishmaniasis is rarely diagnosed in Sweden, and our patients were symptomatic for several months before receiving a final diagnosis. This led us to initiate a clinical investigation in which we tracked the ∼150 patients with ongoing biological treatment who had taken part in the Swedish programme for external rehabilitation at the unit since 2014. Patients who had visited the same rehabilitation centre but who were not treated with biologics (∼50% of all patients) were not tracked; however, information was sent to their clinicians for awareness of cases of leishmaniasis. Until now, three additional patients treated with biological drugs have been diagnosed with cutaneous leishmaniasis caused by *L. infantum*. Another patient with severe RA was admitted to the Department of Infectious Diseases at Sahlgrenska University Hospital with fever, weight loss, pancytopenia and hepatosplenomegaly. The patient had spent 4 weeks at the same rehabilitation unit in Alicante 8 months before the onset of symptoms, had previously received treatment with TNF-α inhibitor during many years, but was presently treated with the monoclonal anti-CD20 antibody rituximab. Clinical investigation revealed *Leishmania* amastigotes in the bone marrow, and molecular typing yielded *L infantum*, confirming the diagnosis of visceral leishmaniasis. The TNF-α inhibitor or anti-CD20 was discontinued as soon as the diagnosis was confirmed. All patients received anti-leishmanial treatment with liposomal amphotericin B. We have an ongoing clinical study for long-term follow-up of this cohort of patients, and further leishmaniasis cases may still be diagnosed. Among the patients not treated with biologics, no case of leishmaniasis has come to our attention so far.

In this report, we want to highlight the importance of awareness of leishmaniasis as a possible and potentially very severe infection in patients treated with anti-CD20 and with anti-TNF-α after travel to endemic regions. Symptomatic disease may occur many months after exposure, which emphasizes the importance of recognition of this infection also in non-endemic countries. The infection may reactivate even after successful treatment, and it is yet unknown whether patients can be treated safely with biological DMARDs ever again after recovery from leishmaniasis. Nevertheless, we do not know whether this observed cluster is an expression of unfortunate chance or is the tip of the iceberg.

*Funding*: No specific funding was received from any funding bodies in the public, commercial or not-for-profit sectors to carry out the work described in this manuscript.

*Disclosure statement*: The authors have declared no conflicts of interest.

## References

[1] Guedes-Barbosa LS, Pereira da Costa I, Fernandes V et al Leishmaniasis during anti-tumor necrosis factor therapy: report of 4 cases and review of the literature (additional 28 cases). Semin Arthritis Rheum 2013;43:152–7. 23777708 10.1016/j.semarthrit.2013.01.006

[2] van Griensven J, Diro E. Visceral leishmaniasis. Infect Dis Clin North Am 2012;26:309–22. 22632641 10.1016/j.idc.2012.03.005

[3] Singh OP, Hasker E, Sacks D, Boelaert M, Sundar S. Asymptomatic *Leishmania* infection: a new challenge for *Leishmania* control. Clin Infect Dis 2014;58:1424–9. 24585564 10.1093/cid/ciu102 PMC4001287

[4] van Griensven J, Carrillo E, López-Vélez R, Lynen L, Moreno J. Leishmaniasis in immunosuppressed individuals. Clin Microbiol Infect 2014;20:286–99. 24450618 10.1111/1469-0691.12556

[5] Zanger P, Gabrysch S. Leishmaniasis in the era of tumor necrosis factor alpha antagonist therapy – a research agenda for Europe. Euro Surveill 2013;18:20542. 23929182 10.2807/1560-7917.es2013.18.30.20542

[6] Gradoni L. Epidemiological surveillance of leishmaniasis in the European Union: operational and research challenges. Euro Surveill 2013;18:20539. 23929176 10.2807/1560-7917.es2013.18.30.20539

[7] Moral L, Rubio EM, Moya M. A leishmanin skin test survey in the human population of l'Alacantí region (Spain): implications for the epidemiology of *Leishmania infantum* infection in southern Europe. Trans R Soc Trop Med Hyg 2002;96:129–32. 12055798 10.1016/s0035-9203(02)90278-6

[8] Söbirk SK, Inghammar M, Collin M, Davidsson L. Imported leishmaniasis in Sweden 1993–2016. Epidemiol Infect 2018;146:1267–74. 29848389 10.1017/S0950268818001309 PMC9134277

